# Post-transcriptional regulation of Profilin-2 by microRNAs and RNA-binding proteins forms a critical regulatory node for early embryonic cell fate decisions

**DOI:** 10.1371/journal.pgen.1012279

**Published:** 2026-09-02

**Authors:** Carolyn Sangokoya, Isabella R. Rosso, Rana Ghandehari-Alavijeh, Robert Blelloch

**Affiliations:** 1 Department of Pathology, University of California, San Francisco, San Francisco, California, United States of America; 2 The Eli and Edythe Broad Center of Regeneration Medicine and Stem Cell Research, Center for Reproductive Sciences, University of California, San Francisco, San Francisco, California, United States of America; 3 Department of Urology, University of California, San Francisco, San Francisco, California, United States of America; Centre de Recherche du CHU de Québec - Université Laval, CANADA

## Abstract

Post-transcriptional control by RNA binding proteins (RBPs) and microRNAs play central roles in mRNA stability and translation, yet how RBPs and microRNAs coordinate in developmental time to regulate cell fate remains poorly understood. Here, we demonstrate that post-transcriptional regulation of the Profilin 2 (Pfn2) transcript is essential for differentiation of embryonic stem cells (ESCs) into the primary germ layer lineages. The Pfn2 3’untranslated region has both an Iron Regulatory Protein binding site (IRE) and a nearby binding site for ESC enriched microRNAs. Deletion of this microRNA site leads to increased PFN2 and reduced FGF signaling during pluripotency transition prior to germ layer formation. In contrast, deletion of the IRE leads to decreased PFN2, a Wnt signaling defect, reduced nuclear beta-catenin, and a subsequent block in mesendodermal lineages during early germ layer formation. We further find that loss of the IRE site results in a cell autonomous defect in Wnt signaling and mesendodermal differentiation. The IRE site acts to stabilize beta-catenin, as disruption of the site leads to reduced nuclear beta-catenin levels. Together, these findings reveal the Pfn2 microRNA-IRE regulatory axis as a critical post-transcriptional regulatory node governing the switch from pluripotency to somatic differentiation.

## Author summary

Embryonic stem cells can give rise to every cell type in the body, but this requires precise regulation of gene expression as development progresses. Although much is known about how genes are turned on and off, less is understood about how cells fine-tune the messenger RNA (mRNA) transcripts to mold the final messages that make proteins. Here, we show that two distinct mRNA regulatory mechanisms cooperate to control expression of Profilin-2 (Pfn2), a gene expressed in early embryonic development. We found that neighboring binding sites for embryonic stem cell-enriched microRNAs and Iron Regulatory Proteins (IRPs) within the Pfn2 mRNA have different functions during development. Loss of the microRNA binding site increases PFN2 levels and disrupts the transition from pluripotency by reducing FGF signaling. In contrast, loss of the IRP binding site decreases PFN2 levels, impairs Wnt/β-catenin signaling, and blocks formation of the mesendoderm embryonic cell lineages. Together, our findings identify Pfn2 as a critical post-transcriptional regulatory node that coordinates successive developmental transitions and reveal how precise regulation of a single mRNA can determine cell fate during early embryonic development.

## Introduction

Post-transcriptional control by RNA binding proteins (RBPs) and microRNAs play central roles in mRNA stability and translation [1]. The Profilin-2 3’untranslated region has a binding site for Iron Regulatory Proteins and a nearby binding site for embryonic stem cell-enriched microRNAs [2–4]. Post-transcriptional regulation of mRNA stability and protein translation is thought to play a central role in cell fate decisions, although only a limited number of examples exist. One such example is the role of the embryonic stem cell-enriched family of microRNAs that promote pluripotency, including the dedifferentiation of somatic cells to induced pluripotent stem cells [2,5,6].

Mouse embryonic stem cells (ESCs) have the remarkable ability to self-renew indefinitely while retaining the potential to make all downstream lineages of the adult body including the germline. To do so, they are typically grown in the presence of LIF and two inhibitors (2i) toward MEK (PD0325901) and GSK3 (CHIR99021) [7]. MEK inhibition blocks FGF signaling while GSK3 inhibition promotes canonical Wnt signaling. GSK3 represses canonical signaling by phosphorylating the secondary messenger beta-catenin which directs it to the proteosome for degradation [8–10]. ESCs grown under these conditions are called naïve ESCs and are transcriptionally similar to the *in vivo* pluripotent epiblast cells of the peri-implantation embryonic day 4.5 (E4.5) blastocyst [11]. Upon removal of LIF and 2i, the naïve ESCs initiate differentiation, first transitioning to a more epithelial-like state resembling the pluripotent epithelial cells of the post-implantation E5.5 egg cylinder stage embryo, also called the formative pluripotent state [12]. The cells can be retained in formative-like state by inhibiting Wnt signaling, providing low level Activin signaling, and allowing for autonomous FGF signaling [13]. Following release of Wnt signaling, these cells than initiate differentiation into somatic germ layers, recapitulating gastrulation *in vivo*. This differentiation can occur spontaneously *in vitro* in the form of embryoid bodies or in more directed fashion in the presence of specific factors [14–16]. These well-defined methods of recapitulating early embryo development in a dish allows for an unprecedented ability to understand fundamental molecular and cellular mechanisms driving cell fate.

MicroRNAs are short non-coding RNAs that function by binding the 3’UTR of target mRNAs, which in turn leads to both destabilization of RNA and inhibition of translation [17–21]. The miR-290 cluster of microRNAs, including miR-291-3p, miR-294, and mir-295, makes up greater than 70% of total microRNAs in ESCs [22]. The miR-290 cluster shares its binding site with the miR-302 cluster, which includes miR-302a-3p, miR-302b-3p, and miR-302d-3p. This binding site (AGCACUU) binds microRNAs miR-291-3p/294-3p/295-3p/302-3p and will be referred to as the miR290/302 site. While expression of the miR-290 cluster dominates ESC pluripotency, peaking in the peri-implantation blastocyst at E4.5, expression of the miR-302 cluster begins with early differentiation in parallel with decreasing miR-290 [23]. Expression of miR-290 and miR-302 clusters overlap from post-implantation to early-mid gastrulation (~E5.5- E7.0), as the miR-290 cluster is downregulated, and miR-302 expression begins and persists until E9.5 [24,25]. Double knockouts of the miR-302/miR-290 cluster microRNAs leads to arrest during peri-gastrulation [23], demonstrating the critical roles of the evolutionarily conserved miR-290 and miR-302 clusters during early mammalian development.

**Fig 1:**
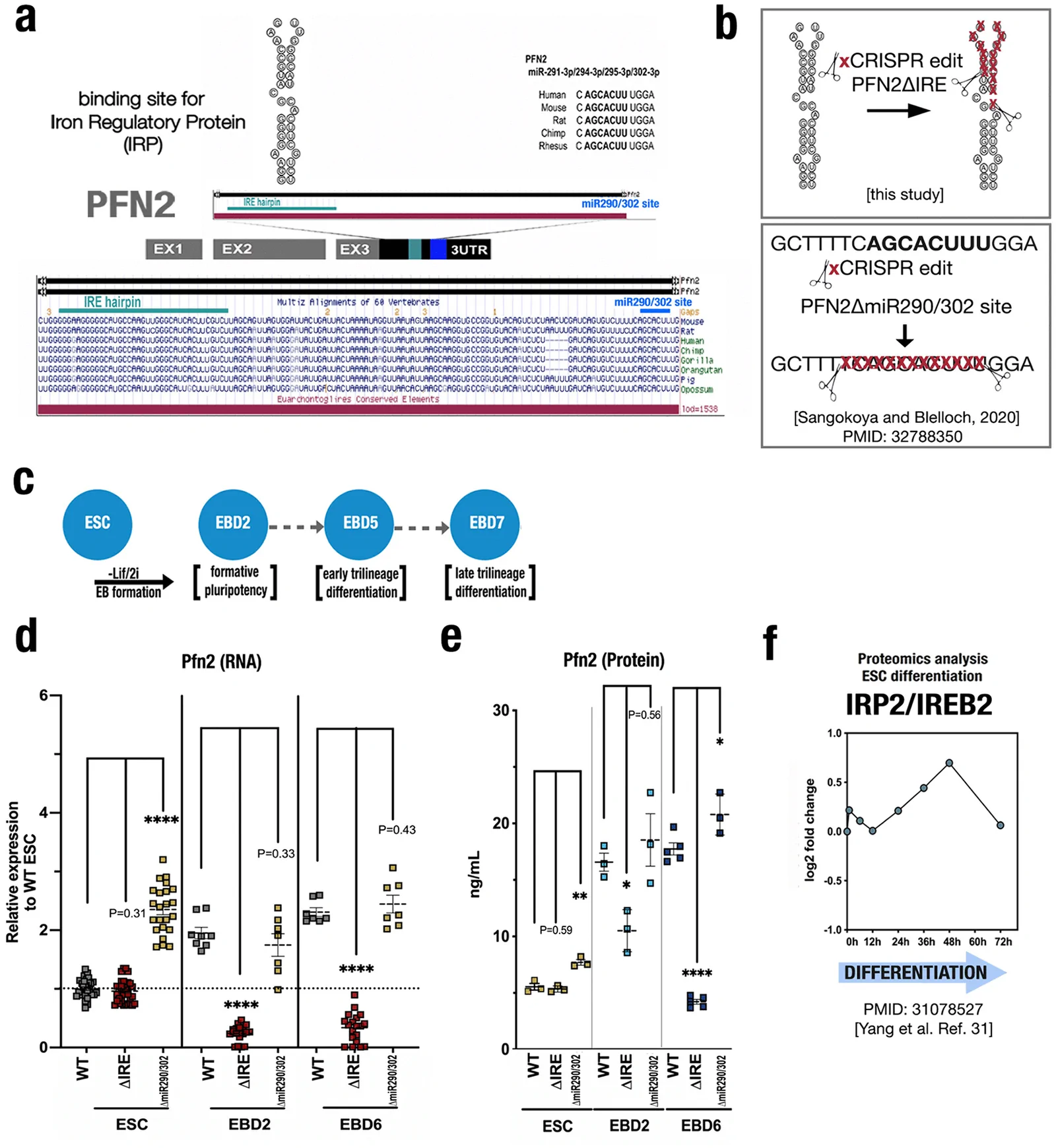
IRP/IRE site regulates PFN2 levels during ESC differentiation. **(a)** Schematic map of PFN2–3’UTR showing locations and evolutionary conservation of the neighboring IRP/IRE and Ago/miR-290 binding sites. **(b)** Representative schematic of CRISPR-edited PFN2–3UTR ∆IRE (top) and PFN2–3UTR ∆miR290/302 (bottom) sequences with red x over deleted sequences **(c)** Schematic of (ESC) differentiation as embryoid bodies (EB) with descriptors of developmental stages at days 2, 5, and 7. **(d)** Expression analysis of Profilin-2 by qPCR in wild-type, PFN2–3UTR ∆IRE, and PFN2–3UTR ∆miR290/302 mutants at naïve pluripotency (ESC): wild-type N = 29; PFN2–3UTR ∆IRE N = 38; PFN2–3UTR ∆miR290/302 N = 23; formative pluripotency (EBD2): wild-type N = 8; PFN2–3UTR ∆IRE N = 19; PFN2–3UTR ∆miR290/302 N = 7;, and early trilineage differentiation (EBD6): wild-type N = 7; PFN2–3UTR ∆IRE N = 19; PFN2–3UTR ∆miR290/302 N = 7 relative to wild-type ESC. Biological replicates for each cell line are shown across independent experiments. Error bars represent SEM. ****P < 0.0001, unpaired two-tailed t test. **(e)** Absolute Profilin-2 protein levels (ng/mL) by ELISA in wild-type, PFN2–3UTR ∆IRE, and PFN2–3UTR ∆miR290/302 mutants at naïve pluripotency (ESC): wild-type N = 3; PFN2–3UTR ∆IRE N = 3; PFN2–3UTR ∆miR290/302 N = 3; formative pluripotency (EBD2): wild-type N = 3; PFN2–3UTR ∆IRE N = 3; PFN2–3UTR ∆miR290/302 N = 3; and early trilineage differentiation (EBD6): wild-type N = 5; PFN2–3UTR ∆IRE N = 5; PFN2–3UTR ∆miR290/302 N = 3; relative to wild-type ESC. Biological replicates for each cell line are shown are shown across independent experiments. Error bars represent SEM. *P < 0.05, **P < 0.01, ****P < 0.0001, unpaired two-tailed t test. **f)** Proteomics analysis result for IRP2 during ESC differentiation reported in Ref [31].

One functional target of the miR-290 cluster in promoting pluripotency is the actin/dynamin binding protein Profilin-2 (PFN2) [2]. Deeper analysis of PFN2’s role has shown that deletion of the only miR290/302 site in the 3’UTR of PFN2 transcript leads to upregulation of PFN2 mRNA and protein levels, which in turn inhibits endocytosis-directed FGF signaling and thus the transition from naïve to formative pluripotency [4]. There is also an iron response element (IRE) in the 3’UTR of PFN2 [3]. This element is less than 100 bp from the miR290/302 site and is conserved through humans (Fig 1a). IREs are target sites for the RNA-binding proteins (RBPs) Iron Response Proteins (IRP) 1 and 2. In contrast to microRNAs, the binding of IRPs to IRE sites in the 3’UTR of a transcript typically results in transcript stabilization [26].

The presence of both a microRNA and an RBP site in close proximity on the 3’ UTR on the PFN2 target transcript raises the question of how they each regulate ESC self-renewal and differentiation. Therefore we deleted the IRE element in the PFN2 3’UTR and compared the consequences to loss of the miR290/302 site. Unlike loss of the miR290/302 site, disruption of the IRE site had no significant impact on PFN2 mRNA and protein levels in naïve ESCs. However, upon differentiation to the formative state and further into the three germ layers, disruption of the IRE element results in a reduction of PFN2 mRNA and protein levels. This reduction results in an inhibition of differentiation toward the three somatic germ layers, especially those of the primitive streak, mesoderm, and endoderm. Additionally, this defect is associated with a decrease in Wnt signaling that is cell autonomous and acts downstream of GSK3β-directed degradation of beta-catenin. There is a decrease in beta-catenin in the nucleus, where it would normally be required to induce the gene expression program of mesendoderm differentiation. Therefore, these data show how the 3’UTR of a single gene can help direct the switch between pluripotency and the trilineage differentiation associated with gastrulation.

## Results

### Divergent post-transcriptional regulation of Profilin-2

To evaluate the role for the *Pfn2* 3’UTR iron response element (IRE) in ESC self-renewal and differentiation, we used CRISPR-Cas9 mutagenesis to delete this IRE site, generating PFN2–3UTR ∆IRE mutant ESCs. These cells were subcloned to produce stable mutant cell lines (Figs 1b and S1a-S1b). These PFN2–3UTR ∆IRE mutant ESCs were compared to matching wild-type control ESCs and previously produced PFN2–3UTR ∆miR290/302 mutant ESCs [4]. When cultured in naïve pluripotency media conditions (LIF + 2i), the PFN2–3UTR ∆IRE mutant ESCs showed no significant difference in PFN2 mRNA and protein levels relative to corresponding wild-type cells (Fig 1b–1d). In contrast, the PFN2–3UTR ∆miR290/302 mutant ESCs showed elevated PFN2 mRNA and protein levels as previously described [4]. To evaluate changes during ESC differentiation, we performed a time course of embryoid body (EB) differentiation (Fig 1b). Upon EB differentiation, wild-type ESCs show an approximately two-fold increase in Profilin-2 mRNA levels and three to four-fold increase in protein levels starting at day 2 (EBD2, representing the naïve to formative pluripotency transition) through day 6 (EBD6, representing germ layer specification) (Fig 1c and 1d). This normal increase in Pfn2 protein with differentiation is significantly decreased in PFN2–3UTR ∆IRE mutant cells (Fig 1e). In contrast, PFN2–3UTR ∆miR290/302 cells show similar levels of PFN2 mRNA and protein as wild-type cells at EBD2 and EBD6. These findings uncover divergent roles for the miR290/302 and IRE sites in the 3’UTR in PFN2. The miR290/302 site suppresses PFN2 levels in naïve ESCs, while the IRE site is essential for the stability of the mRNA and upregulation of PFN2 protein upon ESC differentiation.

The iron regulatory proteins IRP1 and IRP2 are necessary for cellular iron homeostasis and can both bind IRE sites. IRP2 is the dominant of the two IRPs in terms of iron regulation [27] and is downregulated at the protein level when not serving as an RNA-binding regulator of iron homeostasis [26–30]. Recent proteomic analysis during ESC differentiation highlights the peak level of IRP2 48 hours after differentiation, identical to the EBD2 timepoint in our studies [31] (Fig 1f). Additionally, a recent IRP1/2 activity sensor that incorporates the sum of IRP1/2 activity was tested during ESC differentiation and also recapitulates this IRP activity peak at EB2/48 hours differentiation [32].

### Loss of the PFN2–3UTR IRE site inhibits ESC differentiation

**Fig 2:**
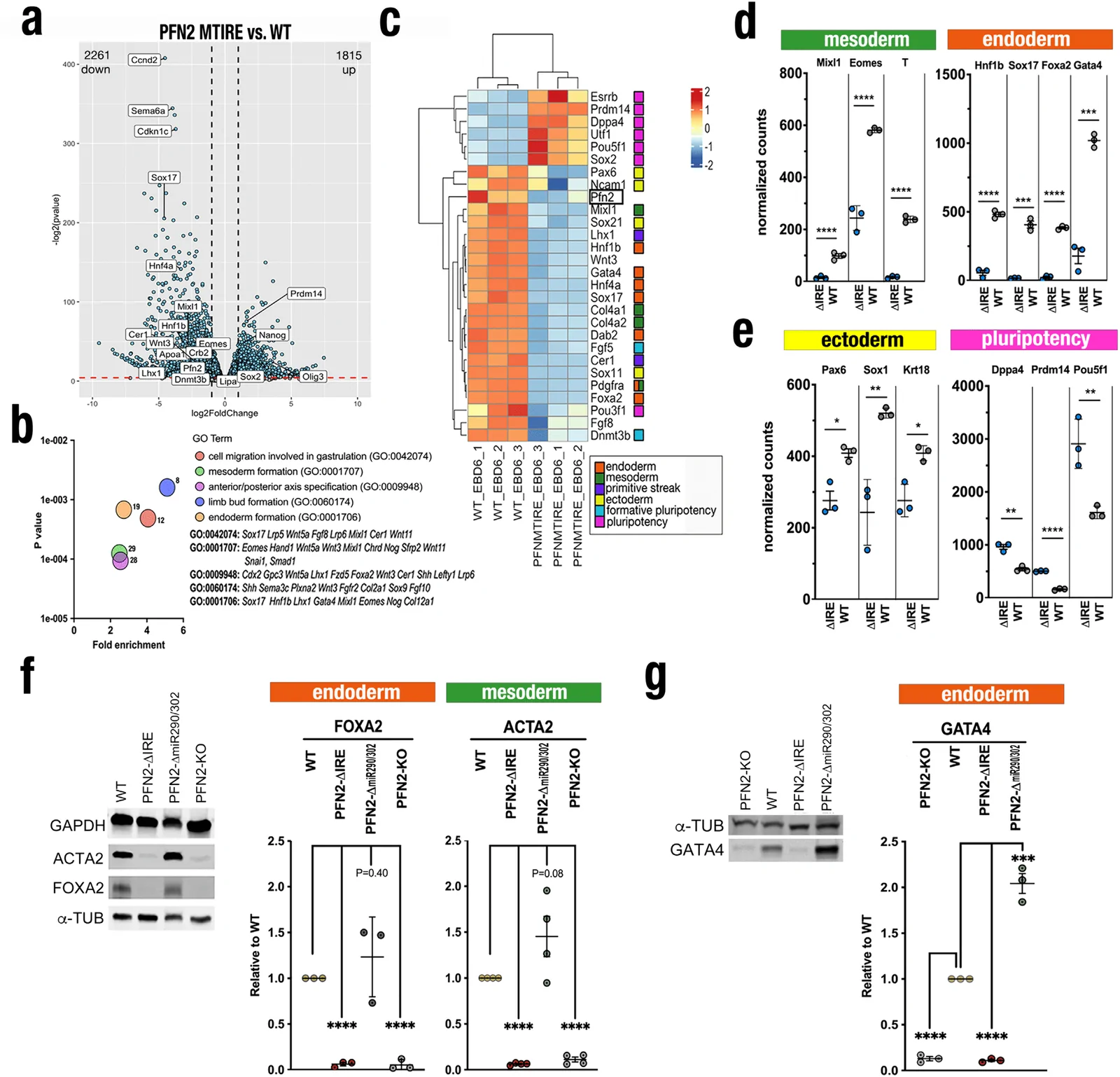
Deletion of IRE in PFN2-3UTR impairs early mesendodermal lineage specification. **(a)** Volcano plot of transcriptomic data PFN2–3UTR ∆IRE versus wild-type at EBD6 showing downregulated (left side) and upregulated (right side) differentially-expressed genes (FDR < 0.05, above red dotted line). For reference, vertical black dotted lines on x axis are present at log2fold change equal to 1. Integration of n = 3 biological replicates for each genotype across independent experiments. **(b)** Gene ontology enrichment analysis of PFN2–3UTR ∆IRE versus wild-type at EBD6. **(c)** Heatmap showing row-wise z-score-scaled normalized gene expression, measured by RNA-seq, of representative pluripotency and trilineage differentiation markers in wild-type and PFN2–3UTR ∆IRE mutants at EBD6; (bottom right) color coding scheme for known developmental and pluripotency markers. endoderm (pink), mesoderm (dark green), primitive streak (dark purple), ectoderm (light green), formative pluripotency (blue), pluripotency (light purple). **(d)** Dot plot of normalized counts of select representative transcripts of mesoderm, endoderm and **(e)** ectoderm, pluripotency-related markers from bulk transcriptomic studies of wild-type and PFN2–3UTR ∆IRE at EBD6. N = 3 biological replicates for each genotype across independent experiments. Error bars represent SEM. *P < 0.05, **P < 0.01, ***P < 0.001, ****P < 0.0001, unpaired two-tailed t test. **(f-g)** Representative immunoblot (left) and immunoblot summary of protein levels (right) of mesoderm (ACTA2) and endoderm (FOXA2, GATA4) markers in wild-type versus PFN2–3UTR ∆IRE, PFN2–3UTR ∆miR290/302, and PFN2-KO mutants at EBD6. Scanned images of unprocessed blots are shown in S2 Fig.

To characterize the impact of the PFN2–3UTR IRE site on the formation of the three germ layers, we performed embryoid body differentiation of wild-type and PFN2–3UTR ∆IRE mutants until EBD6 followed by RNA-seq. Over 4000 genes were dysregulated in the mutants (1815 up, 2261 down, FDR < 0.05) (Fig 2a). Compared to wild-type cells, PFN2–3UTR ∆IRE mutants showed reduced expression of markers for all 3 germ layers including mesoderm (*Mixl1, Col4a1, Col4a2*)*,* endoderm (*Pdgfra, Sox17, Hnf4a, Hnf1b*) and ectoderm (*Sox11, Pax6*), (Fig 2a). In contrast, pluripotency markers were abnormally elevated. Gene ontology analysis showed enrichment for categories such as cell migration involved in gastrulation, endoderm formation, and mesoderm formation (Fig 2b). A closer look at well-described markers of naïve and formative pluripotency and of the three germ layers confirmed elevated expression of naive ESC markers and reduced expression of formative and germ layer markers (Fig 2c). Specifically, naïve-specific markers such as *Esrrb, Dppa4, Utf1,* and *Prdm14* were up, while formative markers such as *Fgf5* and *Dnmt3b* were down. Mesoderm (e.g., *Mixl1, Col4a1, Col4a2, Eomes*, and *T*), endoderm (e.g., *Sox17, Gata4, Hnf4a/b, Foxa2*), and ectoderm markers (e.g., *Pax6, Ncam1, Sox1* and *Krt18*) were consistently down (Fig 2c–2e). qRT-PCR analysis for a subset of these markers confirmed these findings (S2 Fig).

While the miR290/302 site mutants showed reduced expression of the ectoderm marker Pax6, there were normal levels of mesoderm (Mixl1) and endoderm (Gata4, Ttr) markers (S2 Fig). Immunoblot analysis showed that proteins associated with mesoderm (ACTA2) and endoderm (FOXA2, GATA4) were reduced in the IRE site mutant cells at EBD6 (Fig 2f–2g). A similar phenotype was seen with *Pfn2* knockout cells. In contrast, abnormal upregulation of these proteins was seen in the miR290/302 site mutants. Together, these results show a striking defect in ESC differentiation with the loss of the IRE site in the *Pfn2* 3’UTR. In contrast, loss of the miR290/302 site appears to result in a defect in ectoderm differentiation consistent with the prior report of an FGF-related defect in the naïve to formative pluripotency differentiation [4]. Therefore, these two 3’UTR elements have divergent impact on both PFN2 levels and cell fate during ESC differentiation.

### Loss of the PFN2–3UTR IRE site results in a cell autonomous defect in Wnt signaling

**Fig 3:**
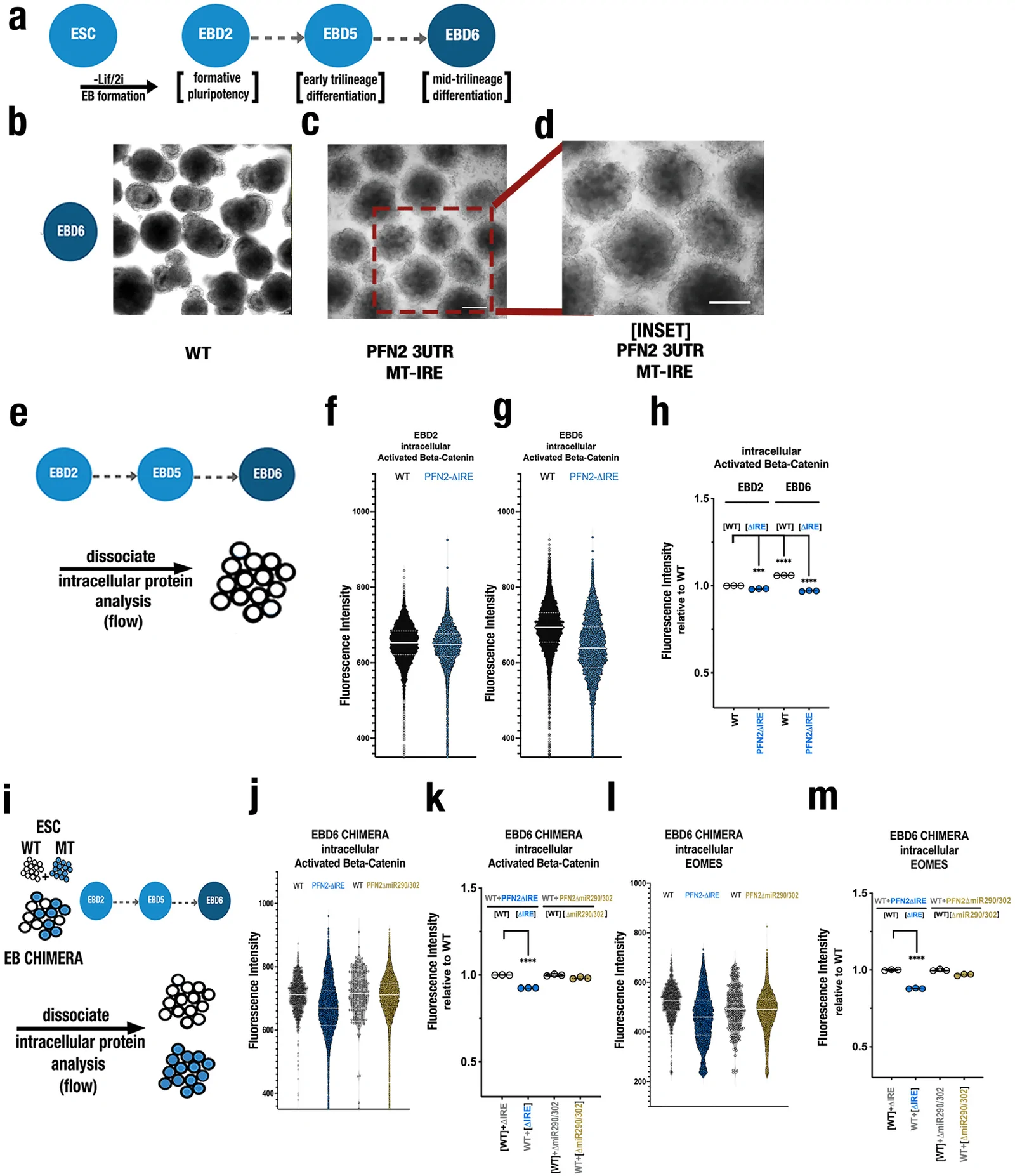
Deletion of IRE inPFN2-3UTR leads to morphologic and molecular defects in embryoid bodies during differentiation. **(a)** Schematic of experimental setup of stem cells (ESC) to embryoid body (EB) formation and differentiation (EBD2–6) with associated developmental trajectory. Representative images of **(b)** wild-type and **(c)** PFN2–3UTR ∆IRE mutant EBD6 embryoid bodies in culture, with **(d)** focus inset highlighting morphologic defect **(e)** Schematic of EB experimental culture and analysis process **(f)** Flow cytometry analysis of dissociated cells from wild-type versus PFN2–3UTR ∆IRE mutant embryoid bodies stained for active (non-phosphorylated) beta-catenin at **(f)** EBD2 and **(g)** EBD6, with **(h)** summary plot of n = 3 independent experiments (single cells of representative experiment shown), ***P < 0.001, ****P < 0.0001, unpaired two-tailed t test. **(i)** Schematic depicting chimera formation and EB time course, intracellular protein analysis. Representative flow cytometry analysis of dissociated **(j)** wild-type versus PFN2–3UTR ∆IRE mutant chimeric embryoid bodies and wild-type versus PFN2–3UTR ∆miR290/302 mutant chimeric embryoid bodies stained for intracellular active beta-catenin at EBD6, with associated **(k)** summary plot of n = 3 independent experiments (single cells of representative experiment shown), ****P < 0.0001, unpaired two-tailed t test. Representative flow cytometry analysis of dissociated **(l)** wild-type versus PFN2–3UTR ∆IRE mutant chimeric embryoid bodies and wild-type versus PFN2–3UTR ∆miR290/302 mutant embryoid bodies stained for intracellular EOMES protein at EBD6, with associated **(m)** summary plot of n = 3 independent experiments (single cells of representative experiment shown), ****P < 0.0001, unpaired two-tailed t test.

We noticed that the PFN2–3UTR ∆IRE mutant EBs were morphologically abnormal compared to wild-type between EBD6 and EBD6.5, showing a loose dissociation at the outer surface of the EBs followed by failure to differentiate or cavitate (Fig 3a–3d). This dysmorphic defect appeared similar to a previously reported morphological defect associated with EB differentiation of ESCs deficient in *Ctnnb1* (beta-catenin) [33]. Beta-catenin plays important roles in both cell adhesion through its interaction with cadherins at cell-cell junctions and in cell signaling as a secondary messenger in canonical Wnt signaling [33,34]. Importantly, Wnt signaling is essential for mesendoderm differentiation during ESC differentiation [35]. Given the combined PFN2–3UTR ∆IRE defects in EB morphogenesis, differentiation into mesoderm and endoderm lineages, we hypothesized a role for increased PFN2 in regulating beta-catenin and enabling differentiation of ESCs into the three germ layers.

Beta-catenin levels are regulated by GSK3β-directed phosphorylation, a post-translational modification that directs beta-catenin to the proteosome for degradation. In the absence of Wnt signaling, beta-catenin is associated with the destruction complex that includes sequential phosphorylation of Thr41, Ser37, and Ser33 by GSK3β [36]. The presence of Wnt-signaling blocks this beta-catenin phosphorylation and degradation [37]. Wnt/β-catenin signaling and subsequent β-catenin/TCF transcriptional activation are specifically mediated through the molecular form of β-catenin that remains unphosphorylated at residues 37 and 41 [38,39], often referred to as the signaling form of β-catenin, or Active β-Catenin (ABC). Therefore, to test our hypothesis, we performed analysis of intracellular unphosphorylated (active) beta-catenin levels at single cell resolution by flow cytometry at EBD2 and at EBD6 (Fig 3e). At EBD2, the intracellular levels of active beta-catenin in PFN2–3UTR ∆IRE mutant EBs were slightly lower than wild-type (~by 1%) while at EBD6, active beta-catenin levels were comparatively much lower (by ~10%) in the PFN2–3UTR ∆IRE mutant cells (Fig 3f–3h). In contrast, active beta-catenin levels were not impacted in PFN2–3UTR ∆miR290/302 mutants at either EBD2 or EBD6 (S3a-S3c Fig).

Next, we asked if the decrease in active beta-catenin was cell autonomous (e.g., cell-intrinsic defects in signaling downstream of the Wnt receptor) or non-autonomous (e.g., non-cell-intrinsic defect upstream of receptor). To do so, we produced chimeric EBs by mixing mutant with wild-type cells at initial 50:50 proportions expressing distinct color labels (S3d Fig). Chimeric EBs composed of both wild-type and PFN2–3UTR ∆IRE showed less of a morphological phenotype than EBs solely made up of PFN2–3UTR ∆IRE cells, although their surfaces still appeared less sharp than WT + WT or WT + PFN2–3UTR ∆miR290/302 chimeric EBs (S3e–S3g Fig). Also, there was skewing of the wild-type cells toward the center of the EBs (S3h–S3j Fig). This observation appears similar to previous observations [40] in embryoid body models that the more adhesive cells will physically concentrate in the center, surrounded by less adhesive cells on the outside, which further support and inform these observations.

Next, we dissociated the EBs into single cells and performed flow cytometric analysis for the color labels, intracellular beta-catenin, and intracellular EOMES as a marker of mesendoderm differentiation (Fig 3i). This analysis showed that the PFN2–3UTR ∆IRE, but not wild-type or PFN2–3UTR ∆miR290/302 cells isolated from the chimeras, showed reduced active and total beta-catenin (Figs 3j, 3k, S3k and S3l). Furthermore, unlike the wild-type or PFN2–3UTR ∆miR290/302 cells, the PFN2–3UTR ∆IRE cells derived from the chimeras failed to upregulate EOMES, (Fig 3l and 3m). Together, these data uncover a cell-autonomous defect in active beta-catenin signaling downstream of the Wnt receptor in the PFN2–3UTR ∆IRE background.

### PFN2–3UTR IRE site functions downstream of GSK3 to stabilize beta-catenin

**Fig 4:**
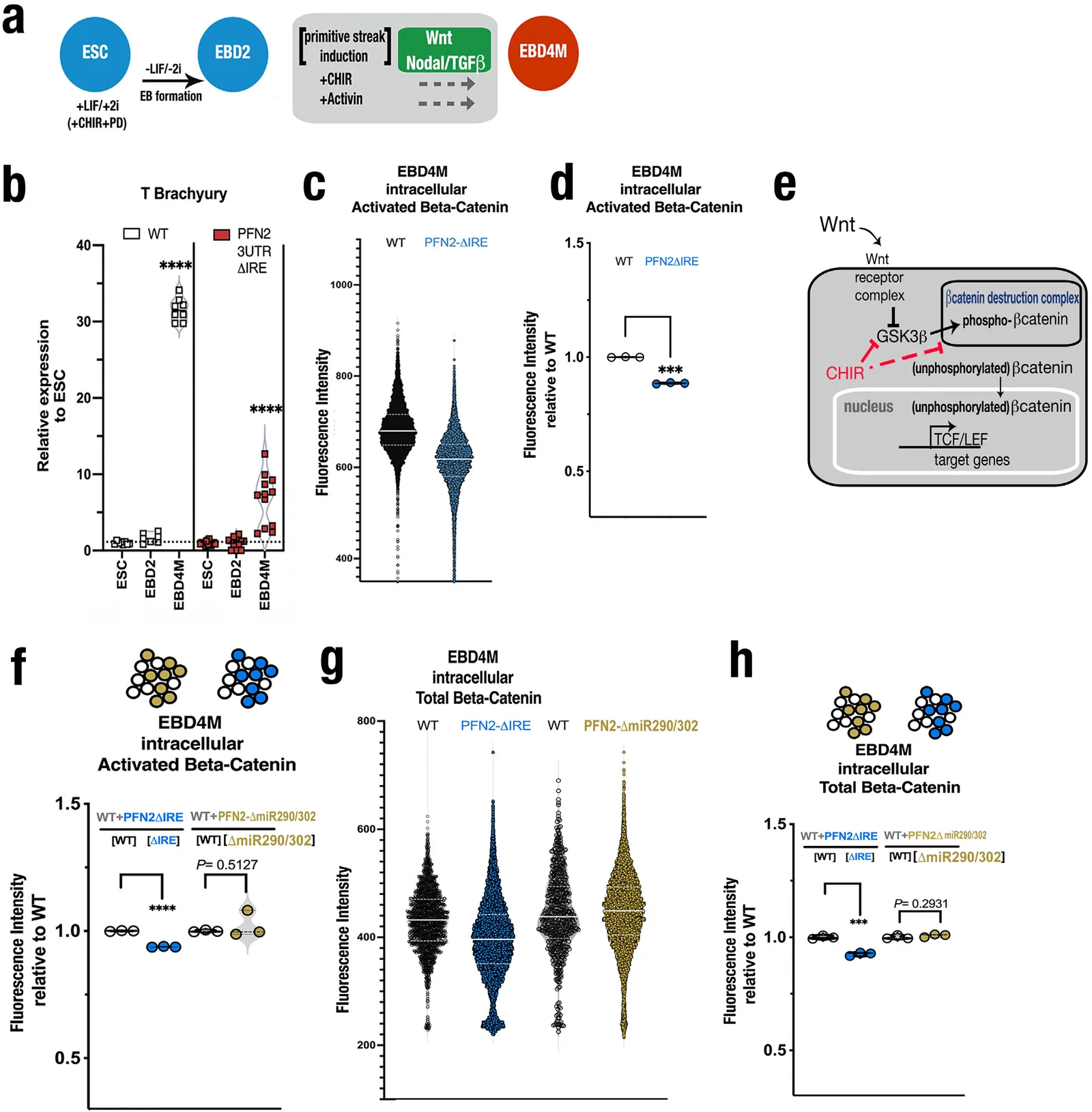
Deletion of IRE in PFN2-3UTR IRE impairs beta-catenin levels and Brachyury expression during primitive streak-like (Wnt/Nodal) induction. **(a)** Schematic experimental setup for directed mesoderm differentiation. **(b)** Relative expression of Brachyury (T) in PFN2–3UTR ∆IRE mutants versus wild-type by quantitative RT-PCR at ESC, EBD2, and EBD4M. N = 3 biological replicates for each genotype across independent experiments. ESC vs EBD4M, **** = P < 0.0001, unpaired two-tailed t test. **(c)** Representative flow cytometry analysis of one experiment of dissociated wild-type versus PFN2–3UTR ∆IRE mutant embryoid bodies stained for active (non-phosphorylated) beta-catenin at EBD4M, with associated **(d)** summary plot of n = 3 independent experiments, ***P < 0.001, unpaired two-tailed t test. Representative flow cytometry analysis of dissociated **(e)** Schematic summary Wnt signaling pathway effect on beta-catenin and upregulation of TCF/LEF target genes (i.e., T Brachyury. **(f)** summary plot of wild-type versus PFN2–3UTR ∆IRE mutant chimeric embryoid bodies (left) and wild-type versus PFN2–3UTR ∆miR290/302 mutant chimeric embryoid bodies (right) stained for active (non-phosphorylated) beta-catenin at EBD4M, **(g)** wild-type versus PFN2–3UTR ∆IRE mutant chimeric embryoid bodies (left) and wild-type versus PFN2–3UTR ∆miR290/302 mutant chimeric embryoid bodies (right) stained for total beta-catenin at EBD4M, (single cells of representative experiment shown), with **(h)** summary of multiple experiments, ***P < 0.001, unpaired two-tailed t test.

To look further into mechanism by which PFN2 impacts beta-catenin and cell differentiation, we transitioned to a directed mesendodermal differentiation protocol (Fig 4a) to perform on wild-type cells and PFN2–3UTR ∆IRE cells. EBs were produced as above, but at day 3, CHIR (CHIR99021, GSK3 inhibitor) and Activin were added to the media to specifically induce mesendoderm differentiation [35], [41–44]. T Brachyury (*Tbxt*) upregulation was used as the readout of successful differentiation into early mesendodermal progenitors [35,43]. T Brachyury is also a direct target of Wnt induced beta-catenin signaling [45]. Wild-type cells showed an over 30x increase in *Tbxt* (Fig 4b). In contrast, PFN2–3UTR ∆IRE cells showed greatly reduced induction (~2x). Intracellular flow cytometry of active beta-catenin remained reduced in PFN2–3UTR ∆IRE relative to wild-type cells even in the presence of the GSK3 inhibitor (Fig 4c and 4d). These data show that even with the GSK3 inhibitor allowing for unphosphorylated beta-catenin to accumulate, there is still a block/impairment where this does not lead to successful T Brachyury upregulation (Fig 4e).

To further confirm the cell autonomous nature of the defect, we repeated the chimera experiments, now under directed mesendodermal culture conditions. Similar to undirected EB differentiation, combining PFN2–3UTR ∆IRE and wild-type cells failed to rescue either active beta-catenin or total beta-catenin levels in directed differentiation conditions (Fig 4f-4h). By contrast, there were no significant differences between wild-type and PFN2–3UTR ∆miR290/302 mutants within chimeras at EBD4M. Together, these results show that PFN2 is acting downstream of GSK3β-directed beta-catenin degradation to regulate Wnt-signaling and thus mesendodermal differentiation.

### PFN2–3UTR IRE regulates nuclear beta-catenin levels

**Fig 5:**
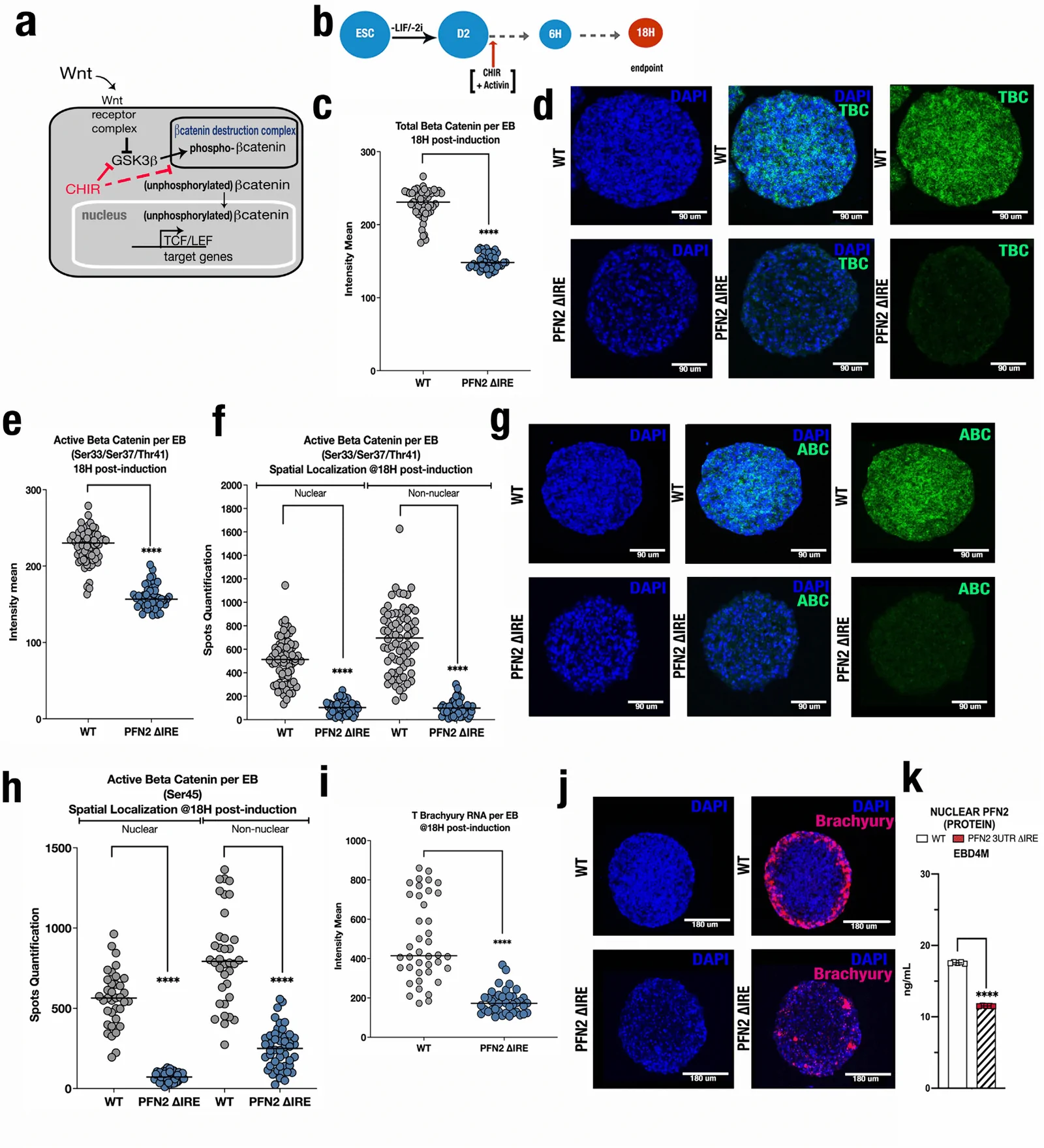
Deletion of IRE in PFN2-3UTR is associated with decreased Profilin2 levels, decreased nuclear active beta-catenin, and failure to induce T Brachyury. **(a)** Schematic of beta-catenin signaling pathway showing cytoplasmic-nuclear transit of beta-catenin following activation leading to TCF/LEF target gene transcription inducing mesendodermal differentiation. **(b)** Schematic experimental setup for time course collection following addition of CHIR+Activin. **(c)** Image analysis, mean intensity of total beta-catenin protein expression per EB at 18 hours post-induction in wild-type (n = 49) and PFN2–3UTR ∆IRE mutant (n = 44) EBs with **(d)** representative analyzed EB images. **(e)** Image analysis, mean intensity of active beta-catenin (Ser33/Ser37/Thr41) protein expression per EB at 18 hours post-induction in wild-type (n = 71) and PFN2–3UTR ∆IRE mutant (n = 49) EBs. **(f)** Image analysis, spot quantification of active beta-catenin (Ser33/Ser37/Thr41) protein expression per EB at 18 hours post-induction in wild-type (n = 71) and PFN2–3UTR ∆IRE mutant (n = 49) EBs with **(g)** representative analyzed EB images. **(h)** Image analysis, spot quantification of active beta-catenin (Ser45) protein expression per EB at 18 hours post-induction in wild-type (n = 36) and PFN2–3UTR ∆IRE mutant (n = 48) EBs. **(i)** Image analysis, mean intensity of *Tbxt* (T Brachyury) RNA transcript expression per EB at 18 hours post-induction in wild-type (n = 40) and PFN2–3UTR ∆IRE mutant (n = 40) EBs with **(j)** representative analyzed EB images. **(k)** Nuclear PFN2 protein by ELISA in EBD4 lysate from wild-type (white) and PFN2–3UTR ∆IRE mutants (red). In graphs c, e, f, h, and i, lines horizontal lines represent mean value, wild-type datapoints are gray, PFN2–3UTR ∆IRE datapoints are blue. Analyzed EBs represent at least n = 3 independent experiments. *P < 0.05, ****P < 0.0001, unpaired two-tailed t test.

Wnt/beta-catenin signaling requires the transport of beta-catenin into the nucleus where it acts as a co-transcription factor in association with TCF/LEF proteins (Fig 5a). Given PFN2’s known association with the cytoskeletal network [46], we hypothesized that destabilization of beta-catenin could be secondary to a failure of beta-catenin transport, retention, or other defect resulting in failed nuclear accumulation. In addition to unphosphorylated Ser33/Ser37/Thr41 (active) beta-catenin, we also wondered if an earlier pre-GSK3 beta-catenin phosphorylation event could be differentially affected in PFN2–3UTR ∆IRE cells. Beta-catenin is phosphorylated at Ser45, an earlier phosphorylation event that primes beta-catenin for subsequent phosphorylation by GSK3β [36], which then phosphorylates beta-catenin at Ser33, Ser37, and Thr41, leading to subsequent destruction by the destruction complex [36]. Unphosphorylated beta-catenin at Ser45 escapes the destruction complex and GSK3β phosphorylation, accumulates in the cytosol, and readily transports into the nucleus. Therefore presence of unphosphorylated beta-catenin protein at Ser45 is another readout of stabilized β-catenin protein functionally active in cell-cell adhesion and mediating transcriptional activity via the Wnt signaling pathway.

To test this hypothesis, we performed directed mesoderm differentiation of wild-type and PFN2–3UTR ∆IRE cells as embryoid bodies by CHIR-Activin induction at EBD2, collecting *in situ* embryoid bodies at 18 hours post-induction (Fig 5b). At this time point, the wild-type and PFN2–3UTR ∆IRE embryoid bodies were paraffin-embedded and processed to assay by RNA in situ hybridization and immunohistochemistry for total beta-catenin, unphosphorylated (active) beta-catenin at Ser33/Ser37/Thr41, unphosphorylated (active) beta-catenin at Ser45, as well as nuclear T Brachyury RNA expression.

Quantitative imaging analysis of wild-type embryoid bodies demonstrate higher (1.5x) total beta-catenin levels per EB compared to PFN2–3UTR ∆IRE (1.10x) EBs (Fig 5c), with representative images shown (Fig 5d). Wild-type embryoid bodies demonstrate higher (1.4x) unphosphorylated Ser33/Ser37/Thr41 active beta-catenin per EB (Fig 5e) compared to PFN2–3UTR ∆IRE EBs (Fig 5e), and 4.8x higher levels in the nuclear fraction (Fig 5f) compared to the nuclear fraction of PFN2–3UTR ∆IRE EBs, with representative images shown (Fig 5g).

Quantitative imaging analysis of wild-type embryoid bodies demonstrate slightly higher (1.10x) Ser45 unphosphorylated active beta-catenin per EB (S4a Fig) compared to PFN2–3UTR ∆IRE EBs, and 7.7x higher levels in the nuclear fraction (Fig 5h) compared to the nuclear fraction of PFN2–3UTR ∆IRE EBs, with representative images shown (S4b Fig). Quantitative imaging analysis of wild-type embryoid bodies demonstrate more abundant (2.7x) nuclear T Brachyury levels per EB compared to PFN2–3UTR ∆IRE, measured by single-molecule probes that amplify signals of individual RNA transcripts (Fig 5i), with representative images shown (Fig 5j). Undifferentiated wild-type and PFN2–3UTR ∆IRE ESCs showed a lower basal level and slight differences in mean intensity of total beta-catenin (wild-type 1.19x higher), and active beta-catenin (wild-type 0.97x lower) (S4c-S4d Fig).

Both quantitatively and visually, the PFN2–3UTR ∆IRE cells demonstrate lower total beta-catenin levels (Fig 5c), with much lower levels of nuclear Ser45 unphosphorylated active beta-catenin (Fig 5h) and of nuclear Ser33/Ser37/Thr41 unphosphorylated active beta-catenin (Fig 5f). Consequentially, the nuclear T Brachyury levels (Fig 5i-5j) phenocopy the results seen in Fig 4. These results indicate a possible defect in transport, retention, or accumulation of nuclear beta-catenin. These data suggest that despite evading degradation by the beta-catenin destruction complex, there is a defect in nuclear accumulation of active beta-catenin and a subsequent effect on induction of T Brachyury, a TCF/LEF target gene essential for mesendodermal differentiation (summarized in S4e Fig).

Finally, we performed a time-course of directed mesendoderm differentiation by CHIR-Activin induction following induction at EBD2, collecting samples at 2, 6, 18, and 48 (EBD4M) hours post-induction and performing nuclear enrichment (S5a‒S5f and S6‒S7 Figs). Nuclear active beta-catenin peaked at 18H in wild-type cells and was ~ 3x fold reduced in PFN2–3UTR ∆IRE cells (S5c and S6 Figs). In contrast, cytoplasmic levels of either active or total beta-catenin were not significantly different between the wild-type and mutant cells (S5d‒S5e Fig). Nuclear T Brachyury levels similarly peaked at 18 hours and were dramatically reduced in PFN2–3UTR ∆IRE cells (S5f Fig). ELISA of PFN2 itself showed reduced levels in both the cytoplasmic and nuclear compartments during the induction time course (S5g Fig) and at the 48 hour (EBD4M) endpoint (Fig 5k). Together, these results further inform the findings described in Fig 4 by qRT-PCR and flow cytometry and in Fig 5b‒5j by biochemical *in situ* RNA and protein analysis, indicating a possible defect in transport, retention, or accumulation of nuclear beta-catenin associated with the reduced levels of PFN2 in PFN2–3UTR ∆IRE cells.

## Discussion

**Fig 6:**
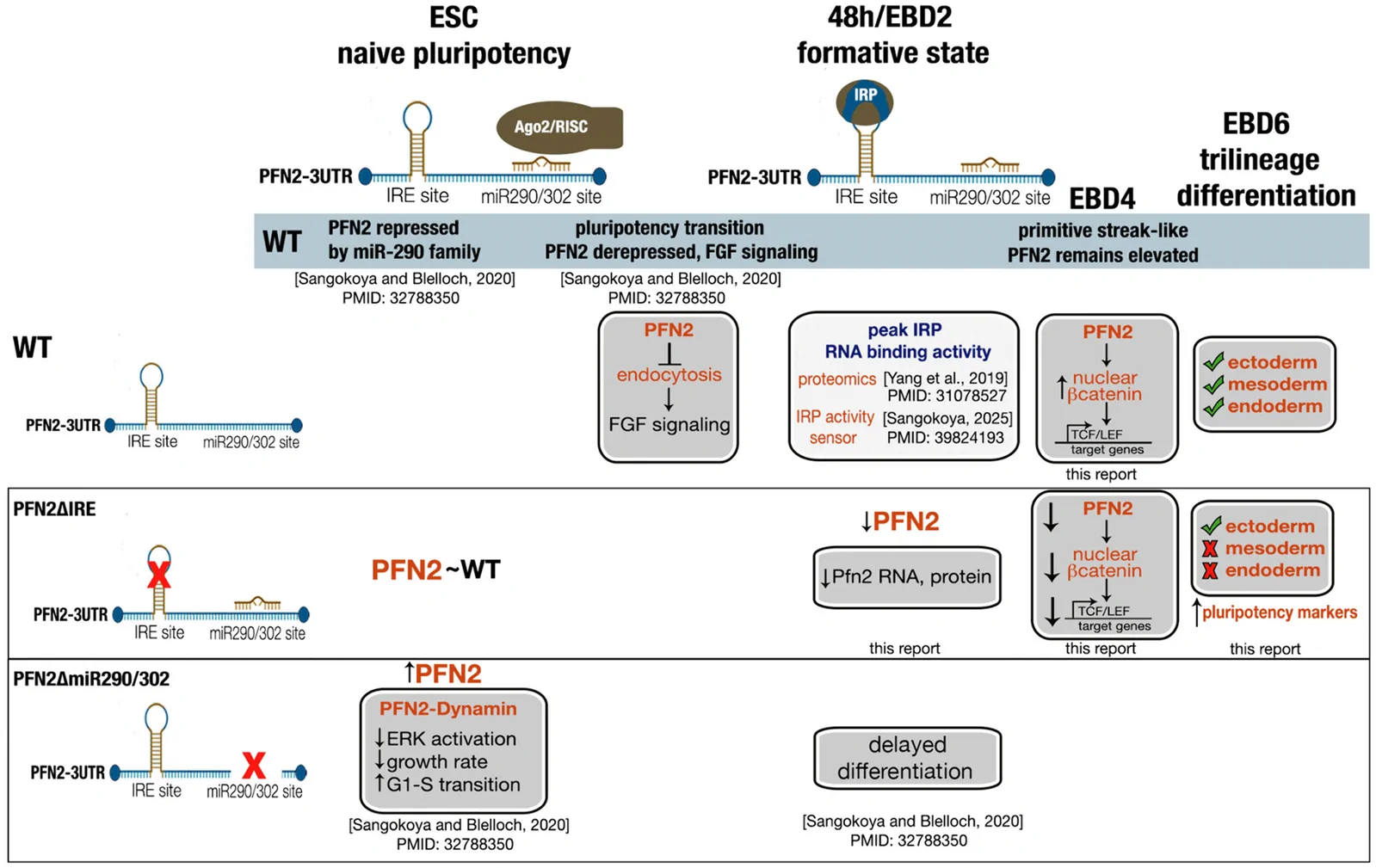
Impact of post-transcriptional control of the Profilin-2 transcript on stem cell biology during pluripotency, pluripotency transition, and early trilineage differentiation. Schematic summary of the impact of Profilin-2 transcript Ago2/miR-290 and IRP/IRE binding sites on stem cell biology during pluripotency, pluripotency transition, and early trilineage differentiation. Clip-art created in BioRender [68].

Together with the recent dissection of the miR290/302 site in the 3’UTR of PFN2 [4], the findings reported here uncover roles for microRNA and an RNA binding protein regulation on the 3’UTR of a single gene during the switch from pluripotency to somatic differentiation (Fig 6). In pluripotent cells, the dominant miR-290 cluster destabilizes PFN2, allowing endocytosis which is essential for normal FGF/ERK signaling [4]. This signaling is important for the normal transition from naïve to formative pluripotency [4], [47–50]. Following the transition, an IRE which is bound by iron response proteins is required for an increase in PFN2 levels, in turn promoting Wnt/beta-catenin signaling. Defects in Wnt/beta-catenin due to deletion of the IRE result in a block in activation of the mesendoderm transcriptional program that normally occurs with gastrulation. The impact of PFN2 on beta-catenin signaling is downstream of GSK driven degradation of the protein and is associated with alterations in the nuclear/cytoplasmic localization of the protein, suggesting a role in the transport, retention, and/or accumulation of beta-catenin in the nucleus. Thus, our findings show how the combinatorial post-transcriptional control of PFN2 plays a critical role in early mammalian cell fate decisions.

Previous examples of microRNA and RBP coordination on a 3’UTR largely involve RBPs enhancing microRNA targeting by improving target site accessibility [51–53] or suppressing microRNA targeting by competitive engagement of overlapping binding sites [53–58]. In the setting of development, for example, the RNA-binding protein Dead end (Dnd1) prohibits the function of several miR-430 and miR-372-family microRNAs by blocking their accessibility to transcript target sites, and this regulation is essential for proper development of primordial germ cells [54]. In the case of PFN2, the RBP and microRNA binding sites are not overlapping, but instead are ~ 100 bases apart and are acting at distinct developmental times. Therefore, it seems unlikely that their opposing impacts on PFN2 levels are due to competitive binding. Instead, these sites reveal a developmentally relevant switch where the predominance of miR-290 microRNA regulation on PFN2 during pluripotency ‘switches’ to a predominant role for IRP binding during somatic differentiation into the three germ layers. We propose that this model could provide a paradigm for other microRNA-RBP pairs playing critical roles in cell fate decisions throughout development.

Our data further shows that PFN2 regulates the transport, retention, or accumulation of beta-catenin in the nucleus. Reduced PFN2 levels during ESC differentiation by deletion of the IRE in its 3’UTR results in reduced nuclear beta-catenin levels without significantly impacting cytoplasmic levels. Nuclear beta-catenin is critical for the differentiation of embryonic stem cells down the mesendoderm lineages [35]. Therefore PFN2’s role in regulating nuclear beta-catenin levels can explain the requirement for elevated PFN2 levels during ESC differentiation into mesendoderm. These findings raise the important question of how PFN2 regulates beta-catenin nuclear import and/or retention. A small number of beta-catenin interacting proteins have been shown to play a role in its nuclear import, but otherwise very little is known [59–61]. Our attempts at co-immunoprecipitation of PFN2 and beta-catenin failed to uncover a direct interaction. Therefore, it remains unclear if the role of PFN2 in beta-catenin cellular localization is direct or indirect. However both PFN2 and beta-catenin interact with actin [62–64] and, therefore, it could be that a direct role for PFN2 in actin control is indirectly impacting beta-catenin nuclear import and/or retention. Indeed, nuclear transport of beta-catenin and actin have been proposed to be tightly intertwined [65]. This actin hypothesis could be tested more directly in future studies. Beta-catenin is a member of the Armadillo family, whose members share the ability to move into the nucleus independent of the classic nuclear localization signal (NLS) which uses an Importin-dependent mechanism for transport [65]. Therefore, it is possible that PFN2 will be found to play important roles in the transport of other Armadillo families as well. In either case, future studies on PFN2’s role in beta-catenin nuclear transport/retention or accumulation are likely identify further novel insights into how the Wnt pathway is controlled in time and space during development.

In summary, our findings reveal that post-transcriptional regulation of the cytoskeletal protein Profilin-2 within a conserved short region of its 3’UTR that includes both an Ago2/microRNA site and an IRP/IRE site is essential for its tight molecular control during early ESC differentiation. This tight control of Profilin-2 expression tunes the FGF/ERK signaling pathway, which is integral to the naïve to formative pluripotency transition, as well as promotes the WNT/Beta-catenin signaling pathway, which is integral to lineage commitment to mesendoderm. This model provides a paradigm for how the coordination of microRNA and RBP binding control on a single transcript over developmental time can regulate cell fate decisions.

### Limitations of study

While this study highlights a previously undescribed developmental switch based on post-transcriptional regulation, there are limitations and unanswered questions. (1) It remains unresolved whether and which extended protein interaction networks interact with PFN2 to modulate nuclear beta-catenin in the context of ESC differentiation. (2) This study is unable to distinguish precisely how PFN2 regulates nuclear beta-catenin – whether by transport, retention, or action on mechanisms directly regulating nuclear accumulation. (3) Of note, in the PFN2–3UTR ∆IRE mutant cells, we appreciated an unexplained slight increase in Pfn2 protein levels at EBD2 in Fig 1e, although still significantly depleted compared to wild-type cells. Other isoforms of the mouse Pfn2 mRNA have been described [66,67] that differ in the last exon and yield protein with different C-terminal amino-acid composition which do not retain the 3′-IRE. Despite these limitations, this study reveals unexplored areas for further investigation. Future studies utilizing parallel and intersectional trans-acting and protein-based approaches to illuminate the spatial context and signaling networks during ESC differentiation would therefore be useful to confirm and extend these findings.

## Methods

### Cell culture and ESC monolayer differentiation

Mouse V6.5 ESCs (RRID:CVCL_C865) were grown and maintained at 37C and 5%CO2 on 0.2% gelatin-coated plates, cultured in ESC medium (custom DMEM) supplemented with 15% FBS (Corning Mediatech), LIF (1000U/mL, custom) and 2i (1uM PD0325901 (Axon MedChem) and 3uM CHIR99021 (Axon MedChem). Differential states of pluripotency from naïve to EpiLC transition were generated by removal of LIF and 2i. In brief, ESCs were plated in ESC medium (as described above). To initiate differentiation, LIF and 2i were removed. Cells were collected at indicated time points following LIF/2i removal.

### Embryoid body generation and differentiation

For embryoid body (EB) generation, ESCs were cultured in rotary (100 rpm) suspension culture on a low attachment plate allowing for self-aggregation and regular reproducible spontaneous differentiation over a course of 7–10 days. EB differentiation was evaluated by quantitative reverse transcriptase PCR, immunoblot analysis, and bulk RNA-sequencing. Directed differentiation to mesendoderm was performed by induction with 10 ng mL^-1^ Activin (GF300, Millipore) and 3 uM CHIR99021 (Axon MedChem). Chimeric embryoid bodies were formed by combining equal numbers of cells prior to rotary suspension culture.

### Quantitative RT -PCR

RNA was extracted from cells using TRIzol (Invitrogen) and quantified on a Nanodrop spectrophotometer (ThermoFisher Scientific). Reverse transcription and quantitative PCR were performed according to manufacturer’s instructions (Maxima, K1641, ThermoScientific; PowerSYBR, Applied Biosystems). Primer sequences are listed in [S1 Table].

### ELISA immunoassay

Sandwich ELISA immunoassay for in vitro quantitative measurement of PFN2. A microplate pre-coated with an antibody specific for Mouse PFN2 was used for sandwich ELISA immunoassay in vitro quantitative measurement of PFN2 (RK06647, ABclonal Science) according to manufacturer’s instructions.

### Generation of site-based mutant cell lines by CRISPR-CAS9 gene editing

Mutant ESC cell lines were generated by cloning gRNAs into a U6 vector (GE100042, Origene) and co-transfection with a Cas9-expressing vector (GE100018, Origene) using Fugene6 (E2691, Roche) as described in [4]. Guide sequences are listed in [S2 Table].

### RNA-seq library preparation and sequencing

RNA (total or poly-adenylated) was extracted using TRIzol (15596026, Invitrogen) or Oligo(dT)25-coated Dynabeads (61002, Invitrogen), respectively. RNA quality was examined by Qubit 2.0 Fluorometer (Life Technologies). cDNA library generation was done using the KAPA RNA Hyperprep kit (KK8541, Roche) following the manufacturer’s instructions. cDNA library quality was assessed using Qubit 2.0 fluorometer to determine concentrations. The 4200 TapeStation system (Agilent) was used to determine insert size following the manufacturer’s instructions. cDNA libraries were then sequenced using the HiSeq 4000 (Illumina) and NovaSeq X Systems (Illumina).

### Bulk RNA-Seq data pre-processing and analysis

Raw fastq files were filtered and preprocessed by quality trimming (-Q33, threshold 20, minimum length 15) using FASTX-Toolkit (http://hannonlab.cshl.edu/fastx_toolkit, [69]). Adapters were removed and reads collapsed using unique molecular identifiers using FASTX-Toolkit and Cutadapt [56]. RNA-seq read mapping and alignment with alignment to mm10 reference genome assembly was performed using STAR [70]. Read count tables were generated using the RSEM program [71]. Differential gene expression was performed using R package DESeq2 [72] for analysis including plotCounts to generate normalized plot counts. R [73] packages including edgeR [74], pheatmap [75] were used for data visualization. FDR was calculated using the Benjamini–Hochberg method. DE genes were defined with FDR less than 0.05 in comparison to the control group. Default settings were used unless otherwise specified.

### Gene ontology and GO enrichment analysis

Gene Ontology reference and GO Enrichment Analysis were accessed and used to perform ontology and fold enrichment analysis using the GO Ontology database https://doi.org/10.5281/zenodo.10536401 [76,77]. Differentially expressed transcripts (with p < 0.05, FDR < 0.05, and log fold change greater than an absolute value of 0.06, for at least a 1.5 fold cutoff) were uploaded to the Gene Ontology Resource at https://geneontology.org. From there, the analysis tool from the PANTHER Classification System [77] is used to perform enrichment analyses by biological process. Results with threshold FDR < 0.05 are reported.

### Immunoblot

Total cell extracts were obtained by lysing cell pellets with ice-cold RIPA lysis buffer containing EDTA-free protease inhibitor mixture (11836170001, Roche) and PhosSTOP phosphatase inhibitor (4906837001, Roche), with DNA shearing by 21-gauge needle. Lysates were incubated at 4C for 15 min and centrifuged at 20,000 x g at 4 C on a table-top centrifuge. The protein concentration of the supernatant was measured by BCA assay, with 30 micrograms of protein resolved by sodium dodecyl sulfate-polyacrylamide gel electrophoresis. After electrophoresis, proteins were transferred onto an Immobilon-FL PVDF membrane (IPFL00010, Millipore) and processed for immunodetection. Primary antibodies were incubated overnight at 4C. Secondary antibodies were incubated for 1h at RT. Images were digitally acquired using a Li-COR Odyssey imager (Li-COR Instruments) and its proprietary software. Antibodies are listed in S4 Table. Bands were quantified using Image J [78].

### Subcellular fractionations

Subcellular fractionations were performed according to manufacturer instructions (78840, Thermo Scientific), and followed by ELISA and/or immunoblot.

### Intracellular staining assay

Cells were first fixed with 4% paraformaldehyde (J61899, ThermoScientific) for 15 min at room temperature, then permeabilized on ice with 0.1% Triton-X (A16046.AE, ThermoScientific) for 5 min and stained for the indicated primary antibody for 30 min at 4C as listed in S4 Table. This was followed by incubation for 30 minutes at 4C with species-specific secondary antibodies as listed in S4 Table. Finally the cells were washed with cold PBS and prepared for analysis by flow cytometry. Antibodies are listed in S4 Table.

### Flow cytometry

After dissociation into single-cell suspension using Trypsin (25200072, ThermoFisher Scientific) or TrypLE (12604039, ThermoFisher Scientific), cells were resuspended in HBSS/1% BSA (14175103, ThermoFisher Scientific; A30075, RPI) and filtered for size by cell strainer (35 micron mesh). Cells were processed by flow cytometry on an LSR II flow cytometer (Becton Dickinson), with events collected by FACS Diva software (BD Biosciences) using a hierarchical gating strategy selecting for a homogenous cell population (forward-scatter area vs. side-scatter area) of single cells (forward-scatter width vs. forward-scatter area). Dot plots and mean fluorescence intensities for gated events were generated using FlowJo analysis software.

### RNA in situ hybridization and immunohistochemistry assays

For RNA in situ hybridization assays, the RNAScope Multiplex v2 Assay kit (ACDBio) measures single-molecule probes that amplify signals of individual RNA transcripts, allowing visualization as distinct dots under a microscope. Images were visualized and captured using an Echo Revolve Fluorescence Microscope. Quantitation of these probes (listed in S3 Table) is interpreted by both intensity levels, number and size of ‘spots’. An integrated workflow includes immunohistochemistry where we are able to concomitantly stain RNA and protein on the same slide for analysis.

### Quantitative imaging analysis

Images were captured using an Echo Revolve Fluorescence Microscope and analyzed using Oxford Instruments Imaris Software (Imaris 11.0.0).

### Statistical analysis

No data were excluded from the analyses. Statistical tests were performed in GraphPad Prism (v10). Data collection and analysis were not performed blind to the conditions of the experiments, but data analyses have been performed with identical parameters and software. Data representation and statistical analyses were also performed using R software. The number of biological replicates and independent experiments, both equal to or greater than 3, is indicated in figure legends. The statistical tests used are indicated in the figure legends.

### Authentication and additional information

V6.5 ES cells and mutant V6.5 ES cells (PFN2–3UTR mutants, PFN2-KO) were authenticated by genotyping (PCR and Sanger sequencing) and by undirected and directed differentiations described in this manuscript. Key experiments were performed within 10 passages under sterile conditions. All cell lines are tested for mycoplasma before use and annually.

## Supporting information

S1 Fig CRISPR editing for PFN2 IRE mutant generation. (TIF)

S2 Fig PFN2 3UTR mutant site effects on ESC trilineage differentiation, uncropped blots Fig 2f and 2g. (TIF)

S3 Fig Decreased beta-catenin and impaired mesoderm specification are cell autonomous defects in cells with disrupted PFN2–3UTR IRE site. (TIF)

S4 Fig Data, images, and summary S5 Fig. (TIF)

S5 Fig Deletion of IRE in PFN2–3UTR is associated with decreased Profilin2 levels, decreased nuclear retention of active beta-catenin, and failure to induce T Brachyury. (TIF)

S6 Fig Uncropped blots from S5a Fig. (TIF)

S7 Fig Uncropped blots from S5b Fig. (TIF)

S1 Table Primer List for qPCR. (TIF)

S2 Table gRNAs sequences. (TIF)

S3 Table RNA probes. (TIF)

S4 Table Antibody List. (TIF)
